# Effects of nursing facility residency on mortality in patients initiating hemodialysis: a retrospective cohort study

**DOI:** 10.1186/s12877-026-07424-8

**Published:** 2026-04-20

**Authors:** Jae Yeong Yoo, Myeon Kyu Cho, Eunjin Bae, Young Youl Hyun, Sungjin Chung, Soon Hyo Kwon, Jang-Hee Cho, Kyung Don Yoo, Woo Yeong Park, In O. Sun, Byung Chul Yu, Gang-Jee Ko, Jae Won Yang, Won Min Hwang, Sang Heon Song, Sung Joon Shin, Yu Ah Hong, Jong-Woo Yoon, Hyunsuk Kim

**Affiliations:** 1 Department of Internal Medicine, Hallym University Medical Center, Chuncheon Sacred Heart Hospital, Sakjuro 77, Chuncheon, Gangwondo, Chuncheon, 24253 Republic of Korea; 2 Division of Nephrology, Department of Internal Medicine, Gyeongsang National University College of Medicine, Jinju, Republic of Korea; 3 Division of Nephrology, Department of Internal Medicine, Kangbuk Samsung Hospital, Sungkyunkwan University School of Medicine, Seoul, Republic of Korea; 4 Division of Nephrology, Department of Internal Medicine, Yeouido St. Mary’s Hospital, College of Medicine, The Catholic University of Korea, Seoul, Republic of Korea; 5 Division of Nephrology, Department of Internal Medicine, Soonchunhyang University Seoul Hospital, Seoul, Republic of Korea; 6 Division of Nephrology, Department of Internal Medicine, Kyungpook National University Hospital, School of Medicine, Kyungpook National University, Daegu, Republic of Korea; 7 Division of Nephrology, Department of Internal Medicine, Ulsan University Hospital, University of Ulsan College of Medicine, Ulsan, Republic of Korea; 8 Division of Nephrology, Department of Internal Medicine, Keimyung University Dongsan Hospital, Keimyung University School of Medicine, Daegu, Republic of Korea; 9 Division of Nephrology, Department of Internal Medicine, Presbyterian Medical Center, Jeonju, Republic of Korea; 10 Division of Nephrology, Department of Internal Medicine, Soonchunhyang University Bucheon Hospital, Bucheon, Republic of Korea; 11 Division of Nephrology, Department of Internal Medicine, Korea University Guro Hospital, Korea University College of Medicine, Seoul, Republic of Korea; 12 Division of Nephrology, Department of Internal Medicine, Yonsei University Wonju College of Medicine, Wonju, Republic of Korea; 13 Division of Nephrology, Department of Internal Medicine, Konyang University Hospital, Daejeon, Republic of Korea; 14 Division of Nephrology, Department of Internal Medicine and Biomedical Research Institute, Pusan National University Hospital, Busan, Republic of Korea; 15 Division of Nephrology, Department of Internal Medicine, Dongguk University Ilsan Hospital, Dongguk University School of Medicine, Goyang, Republic of Korea; 16 Division of Nephrology, Department of Internal Medicin, Daejeon St. Mary’s Hospital, College of Medicine, The Catholic University of Korea, Seoul, Republic of Korea

**Keywords:** Chronic kidney disease, Hemodialysis, Mortality, Nursing facility, Older adults

## Abstract

**Background:**

As the population of older adults grows and nuclear families become increasingly prevalent, the number of older adults cared for in nursing facilities is rising. We investigated whether undergoing dialysis while residing in a nursing facility impacts mortality rates among dialysis patients.

**Methods:**

We enrolled 2,597 patients who visited hemodialysis clinics at 17 teaching hospitals and collected data on underlying diseases, laboratory findings, and medications, comparing patients who initiated dialysis during residency in a nursing facility with those who did not. Multivariate survival analysis was then performed to determine whether initiating dialysis while in a nursing facility increased mortality.

**Results:**

Of the 2,597 patients, 9.1% (*n* = 237) initiated dialysis while residing in a nursing facility. Compared with community-dwelling patients, facility residents were older, more often female, and had a higher burden of comorbidities including diabetes, dementia, and cardiovascular disease, along with lower markers of nutritional status. In multivariable Cox regression analysis adjusting for demographic factors, comorbidities, laboratory parameters, and medication use, nursing facility residency at dialysis initiation was independently associated with higher mortality (adjusted HR 1.66, 95% CI 1.30–2.22), corresponding to an approximately 66% increased risk of death.

**Conclusions:**

In summary, the current study suggests a correlation between starting dialysis in a nursing facility and a higher mortality rate among dialysis patients.

## Background

The number of patients receiving hemodialysis (HD) in Korea continues to rise as the population ages, with older adults now accounting for more than half of the HD population [1, 2]. According to the 2024 annual report of the Korean Renal Data System (KORDS) published by the Korean Society of Nephrology (KSN) [3], the number of patients with end-stage renal disease (ESRD) receiving HD has steadily grown since the 1980s, reaching 137,705 in 2023. Although overall survival of HD patients has improved, mortality remains disproportionately high among older patients, particularly those aged 75 years and older. In parallel with population aging, the number of older adults residing in nursing facilities in Korea has increased substantially in recent years [4].

Recently, Chen et al. reported that dialysis patients with a history of nursing facility stays, regardless of duration, exhibited an adjusted hazard ratio of nearly 2.4 for standardized mortality ratio [5]. Thus, in addition to previously established risk factors contributing to elevated mortality rates among older HD patients—such as infections, malignancies, cerebrovascular diseases (CVD) [6, 7], frailty [8], falls [9], or functional and cognitive impairment [10]— residence in nursing facilities may be associated with increased mortality. Recognizing this could guide new strategies aimed at reducing the notably high mortality rates in this population.

As the population of older adults continues to grow and nuclear families increasingly predominate, the number of older individuals residing in nursing facilities and requiring specialized care is expected to rise. Given this context, we investigated whether initiating dialysis while residing in a nursing facility influences mortality rates among HD patients.

## Methods

### Definition of nursing facility

In this study, “nursing facility” refers to long-term care institutions for older adults certified under the Korean Long-Term Care Insurance (LTCI) system. These facilities provide sustained assistance with activities of daily living and supervision for individuals with significant functional limitations, and are distinguished from acute care hospitals or short-term rehabilitation units. Institutional care under LTCI includes facility-based residential care settings regulated by the LTCI Act and related welfare legislation, with eligibility determined by standardized functional assessments for long-term care needs. Residents of these institutions receive regular nursing and personal care support as part of the national insurance benefit framework.

Under Korean regulatory practice, skilled nursing units in such facilities are required to maintain formal nursing staffing, typically with at least one registered nurse or licensed practical nurse per six residents, and at least 50% of these licensed nursing staff being registered nurses, with visiting physicians conducting periodic clinical oversight; other healthcare professionals may be involved depending on local staffing arrangements and care needs. Group homes or other small congregate living arrangements reported in national statistics were not included as “nursing facilities” in this study unless they met criteria for institutional long-term care under the LTCI system and were documented as the resident’s primary residence at the time of dialysis initiation.

### Study population

**Fig. 1:**
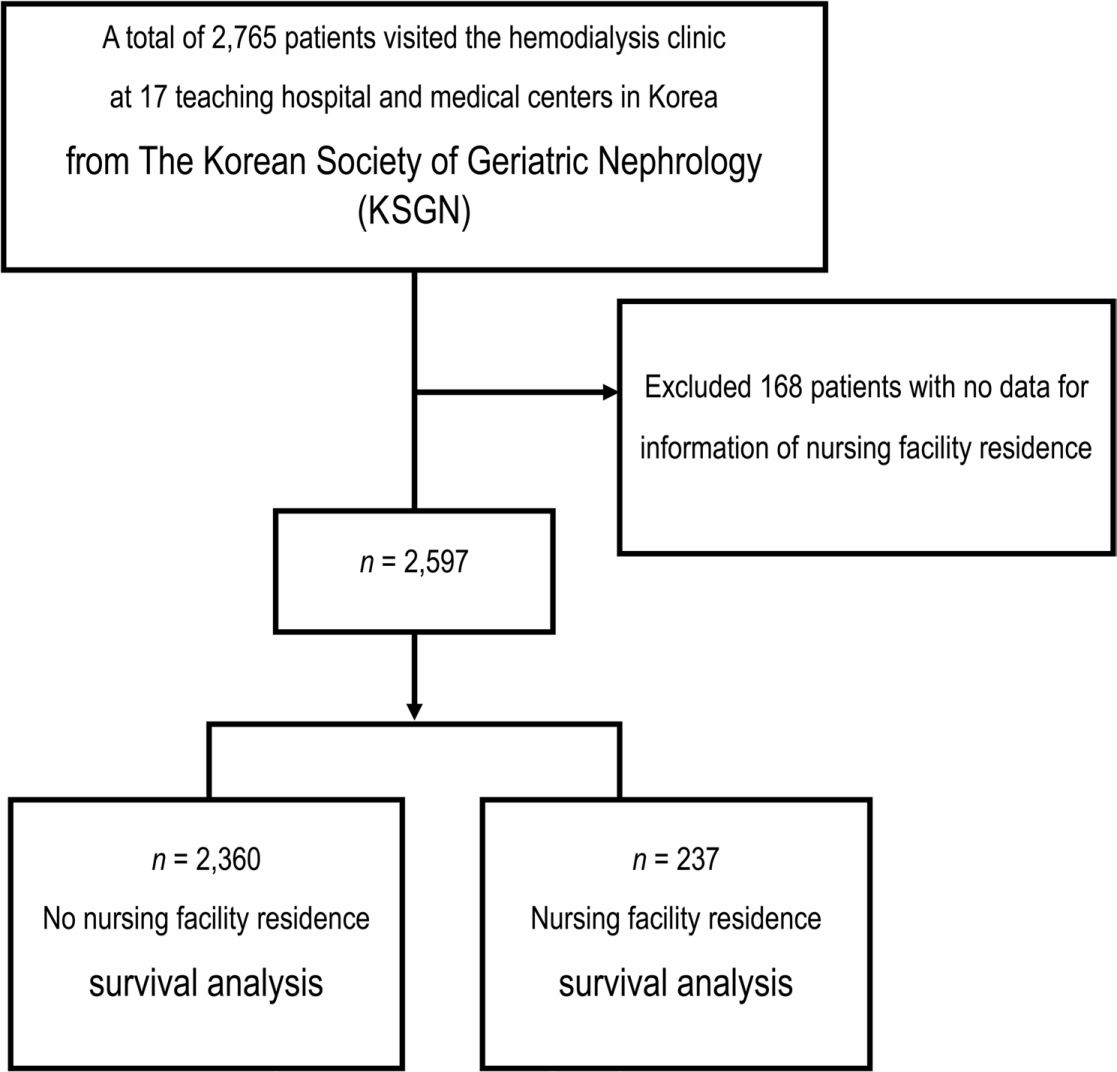
Study design and patient selection flowchart. A total of 2,765 patients who visited the hemodialysis clinics at 17 teaching hospitals and medical centers in Korea, enrolled through the Korean Society of Geriatric Nephrology, were initially surveyed. After excluding 168 patients with missing data regarding nursing facility residency, 2,597 patients were included for analysis. We compared the clinical characteristics, including underlying diseases, laboratory findings, and medication use, between patients who initiated dialysis while residing in a nursing facility and those who did not. Subsequently, multivariate survival analysis was conducted to evaluate whether initiating dialysis during nursing facility residency influenced patient mortality

This study analyzed data from 2,765 HD patients aged 70 years or older who were enrolled by the Korean Society of Geriatric Nephrology (KSGN) from January 1, 2010, to December 21, 2017. The included 2,765 patients had medical records containing the following information: age, sex, weight, cause of ESRD, preparation for dialysis – defined as having undergone vascular access surgery prior to dialysis initiation, initial dialysis access type, maintenance dialysis access type, dementia, malignancy, and comorbidities (diabetes mellitus [DM], lymphoma, ischemic heart disease, PAD, CVD, heart failure [HF], atrial fibrillation [AF], hypertension, liver cirrhosis, rheumatoid arthritis, and fracture). After excluding 168 patients who lacked information regarding nursing facility residence, the remaining 2,597 patients were divided into two groups based on whether they had a history of nursing facility residency prior to initiating dialysis: 237 patients had resided in a nursing facility, and 2,360 patients had not (Fig. 1).

### Study design and follow-up

This multicenter retrospective cohort study defined the index date (time zero) as the date of the first hemodialysis session for each patient. The primary exposure, nursing facility residency, was strictly defined as the patient’s residence status at the time of dialysis initiation (baseline status); due to the limitations of the retrospective dataset, subsequent changes in residence during the follow-up period were not analyzed, and the exposure was treated as a time-fixed variable defined at baseline and no reclassification occurred during follow-up, precluding immortal time bias related to post-baseline exposure changes. Patients were followed up from the index date until death or the study censoring date of December 31, 2020, whichever occurred first.

### Data collection and covariates

After dividing patients into these groups, we statistically analyzed ejection fraction (EF), laboratory findings—including complete blood count, intact parathyroid hormone (iPTH), blood urea nitrogen (BUN), creatinine, albumin, alkaline phosphatase (ALP), phosphorus, and total cholesterol (Table 2)—and medication use (Table 3). Mortality data up to December 31, 2020, were provided by the Korean National Statistical Office [12]. Laboratory data were collected within 1 month prior to dialysis initiation (pre-dialysis values). Echocardiographic data, including EF, were obtained from the most recent echocardiogram performed as part of routine pre-dialysis evaluation within 6 months prior to dialysis initiation. For measuring EF, we used Biplane Simpson’s Method [11]. Medication data were comprehensively extracted from the electronic medical record (EMR) of each participating hospital at the time of dialysis initiation. All patients were under care of nephrologists at the time of dialysis initiation. Data were extracted from institutional electronic medical records by trained personnel. Baseline variables were defined using standardized clinical documentation. Data completeness was verified prior to analysis.

All study methods were carried out in accordance with applicable guidelines and regulations. Patients’ clinical data were collected after receiving approval from the Institutional Review Board (IRB) for each study period and in accordance with the principles outlined in the Declaration of Helsinki. Informed consent was waived by each IRB, and personally identifiable information was adequately protected. Informed consent was specifically waived by the IRBs of the following institutions: Korea University Guro Hospital, Soonchunhyang University Seoul Hospital, The Catholic University of Korea Incheon St. Mary’s Hospital, Hallym University Chuncheon Sacred Heart Hospital, Keimyung University Dongsan Hospital, Gyeongsang National University Changwon Hospital, Presbyterian Medical Center, Dongguk University Ilsan Hospital, Wonju Severance Christian Hospital, Ulsan University Hospital, Soonchunhyang University Bucheon Hospital, Yeouido St. Mary’s Hospital, Kyungpook National University Hospital, Pusan National University Hospital, Kangbuk Samsung Hospital, Konyang University Hospital, and Daejeon St. Mary’s Hospital. The IRB numbers for each institution involved in this study are provided in an article published in *BMC Nephrology* [12].

### Statistical analysis

Continuous and nominal variables are expressed as means and standard deviations. Variables with a normal distribution were analyzed using the Student t-test, independent two-sample t-test, and analysis of variance. Variables with a non-normal distribution were analyzed using the Wilcoxon rank-sum test. Categorical variables are presented as frequencies and percentages, and comparisons were made using the chi-square test or Fisher exact test. The Kaplan–Meier survival curve was employed to evaluate differences in survival rates between the two patient groups. Cox proportional hazards models were used to examine mortality associated with nursing facility residency. We performed univariable Cox regression analyses and selected covariates for multivariable models based on both statistical significance and established clinical relevance; a two-sided p-value < 0.05 was considered statistically significant. Multicollinearity was evaluated using the variance inflation factor (VIF), and all variables demonstrated VIF values < 2.0, indicating no substantial collinearity. In addition, even when statistical multicollinearity was not evident, clinically redundant variables were reduced to a single representative variable. Hazard ratios for continuous variables were estimated per unit increase based on the original measurement scale of each variable. Analyses were conducted using complete cases; no imputation was performed for missing data; complete-case exclusion rate was 6.1%, 168 of 2,765 patients. All statistical analyses were performed using SPSS software (version 21; IBM, Illinois).

## Results

The baseline demographic and medical characteristics of patients according to nursing facility residency are provided in Table 1. Among the total of 2,597 patients, 9.1% (*n* = 237) initiated dialysis while residing in nursing facilities. Compared with patients who had no history of nursing facility residency, those who resided in nursing facilities were slightly older (*p* = 0.002), had a higher proportion of women (*p* < 0.001), and accordingly had lower average body weight (*p* < 0.001). There were no statistically significant differences in primary etiology (*p* = 0.059), preparation for dialysis (*p* = 0.068), or dialysis access type at initiation (*p* = 0.446) between the two groups. However, patients with a history of nursing facility residency had significantly higher prevalence rates of several comorbidities, including dementia (*p* < 0.001), malignancy (*p* = 0.042), cerebrovascular disease (CVD; *p* < 0.001), heart failure (HF; *p* = 0.003), atrial fibrillation (AF; *p* = 0.003), and diabetes mellitus (DM; *p* = 0.022). The incidence of fractures before initiating HD was also higher in the nursing facility group (*p* = 0.009).

**Table 1:** Demographic information and medical history of patients according to nursing facility residency

Variables	No nursing facility (*n* = 2360)	Nursing facility (*n* = 237)	*P* value	SMD
Age, years, mean ± SD	77.82 ± 5.52	78.96 ± 5.42	0.002	0.21
Sex, male, n (%)	1341 (56.8%)	97 (40.9%)	< 0.001	0.32
Weight, kg, mean ± SD	59.02 ± 10.93	55.90 ± 11.46	< 0.001	0.28
Primary etiology, n (%)			0.059	
DKD	1114 (47.2%)	131 (55.3%)		0.16
GN	156 (6.6%)	17 (7.2%)		0.02
Renal vascular	576 (24.4%)	52 (21.9%)		0.06
Other	514 (21.8%)	37 (15.6%)		0.16
Preparation for dialysis	556 (23.6%)	54 (22.8%)	0.068	0.02
Access when starting dialysis, n (%)			0.446	
Catheter	1937 (82.1%)	200 (84.4%)		0.06
AVF	86 (3.6%)	10 (4.2%)		0.03
AVG	337 (14.3%)	27 (11.4%)		0.08
Dementia	81 (4.8%)	32 (18.3%)	< 0.001	0.57
Malignancy, n (%)			0.042	
No	1969 (83.4%)	213 (89.9%)		0.18
Cured	333 (14.1%)	23 (9.7%)		0.13
On treatment	31 (1.3%)	1 (0.4%)		0.08
On conservative care	27 (1.1%)	0 (0.0%)		
Lymphoma, n (%)	21 (0.9%)	3 (1.3%)	0.565	0.04
Ischemic heart disease, n (%)	503 (21.3%)	57 (24.1%)	0.329	0.07
Peripheral artery disease	155 (6.6%)	22 (9.3%)	0.114	0.10
(resting pain or confirmed by angiography), n (%)				
Cerebrovascular disease, yes, n (%)	452 (19.2%)	77 (32.5%)	< 0.001	0.33
Heart failure, n (%)	410 (17.4%)	60 (25.3%)	0.003	0.21
Atrial fibrillation, n (%)	217 (9.2%)	36 (15.2%)	0.003	0.20
Diabetes, n (%)	1350 (57.3%)	154 (65.0%)	0.022	0.16
Hypertension, n (%)	2107 (89.3%)	205 (86.9%)	0.257	0.08
Liver cirrhosis, n (%)	77 (3.3%)	7 (3.0%)	0.798	0.02
Rheumatic disease, n (%)	198 (8.4%)	16 (6.8%)	0.379	0.06
Fracture before hemodialysis, n (%)			0.009	0.19
No history of fracture	2185 (92.6%)	207 (87.3%)		
Hip fracture within 1 year	88 (3.7%)	17 (7.2%)		0.18
Vertebral compression fracture within 1 year	29 (1.2%)	7 (3.0%)		0.13
Other fracture within 1 year	58 (2.5%)	6 (2.5%)		0.00

*Abbreviations*: *SMD* Standardized mean difference, *SD* Standard deviation, *DKD* Diabetic kidney disease, *GN* Glomerulonephritis, *AVF* Arteriovenous fistula, *AVG* Arteriovenous graft

As presented in Table 2, EF and laboratory findings were compared between the two groups. Although EF did not significantly differ between groups (*p* = 0.284), white blood cell count (*p* = 0.002) and ALP (*p* < 0.001) levels were higher in patients with a nursing facility history. Importantly, patients with nursing facility residency exhibited lower lymphocyte counts (*p* = 0.026), iPTH (*p* = 0.009), serum albumin (*p* < 0.001), phosphorus (*p* = 0.003), and total cholesterol levels (*p* = 0.02), indicating a relatively poorer nutritional status. Regarding medications, as shown in Table 3, there were mostly no significant differences between the groups, with some exceptions. The proportion of patients receiving antiplatelet agents (*p* < 0.001), warfarin (*p* = 0.038), antidepressants (*p* = 0.001), and dementia medications (*p* < 0.001) was significantly higher among patients with nursing facility residency.

**Table 2:** Ejection fraction and laboratory findings of patients according to nursing facility residency

Variables	No nursing facility (*n* = 2360)	Nursing facility (*n* = 237)	*P* value
Ejection fraction, %, mean ± SD	58.18 ± 11.05	57.27 ± 11.88	0.284
WBC, /mm^3^, mean ± SD	9109.54 ± 11769.23	12595.72 ± 39333.02	0.002
Neutrophil count, /mm^3^, mean ± SD	72.16 ± 12.74	73.09 ± 13.00	0.664
Lymphocyte count, /mm^3^, mean ± SD	18.05 ± 9.65	15.04 ± 7.60	0.026
Hb, g/dL, mean ± SD	9.22 ± 2.19	9.29 ± 1.77	0.625
PLT count, /mm^3^, mean ± SD	177512.75 ± 93659.18	175470.79 ± 90407.93	0.749
iPTH, pg/mL, mean ± SD	189.72 ± 178.67	152.54 ± 147.62	0.009
BUN, mg/dL, mean ± SD	79.71 ± 34.22	76.04 ± 37.55	0.12
Creatinine, mg/dL, mean ± SD	6.81 ± 14.24	5.50 ± 2.78	0.157
Albumin, g/dL, mean ± SD	3.36 ± 0.60	3.11 ± 0.62	< 0.001
ALP, IU/L, mean ± SD	139.87 ± 128.92	198.95 ± 192.96	< 0.001
Phosphorus, mg/dL, mean ± SD	4.97 ± 1.88	4.57 ± 1.82	0.003
Total cholesterol, mg/dL, mean ± SD	143.11 ± 47.93	134.97 ± 44.95	0.02

*Abbreviations*: *SD* Standard deviation, *WBC* White blood cell, *Hb* hemoglobin, *PLT* Platelet, *iPTH* intact parathyroid hormone, *BUN* Blood urea nitrogen, *ALP* Alkaline phosphatase

**Table 3:** Medication of patients according to nursing facility residency

Variables	No nursing facility	Nursing facility	*P* value
RAAS blockade, n (%)	1190 (50.4%)	113 (47.7%)	0.421
Beta blocker, n (%)	923 (39.1%)	82 (34.6%)	0.173
Calcium channel blocker, n (%)	1405 (59.5%)	130 (54.9%)	0.162
Alpha blocker, n (%)	262 (11.1%)	18 (7.6%)	0.096
Diuretics, n (%)	1373 (58.2%)	130 (54.9%)	0.319
Statin, n (%)	921 (39.0%)	84 (35.4%)	0.28
Antiplatelet agent, n (%)	1185 (50.3%)	152 (64.4%)	< 0.001
Warfarin, n (%)	85 (3.6%)	15 (6.3%)	0.038
Calcium-based phosphate binder, n (%)	892 (37.8%)	81 (34.2%)	0.272
Sevelamer, n (%)	86 (3.6%)	14 (5.9%)	0.084
Lanthanum, n (%)	16 (0.7%)	2 (0.8%)	0.769
Depression drug, n (%)	173 (8.5%)	34 (15.5%)	0.001
Dementia drug, n (%)	77 (3.3%)	34 (14.3%)	< 0.001

*Abbreviations*: *RAAS* Renin-angiotensin-aldosterone system

**Fig. 2:**
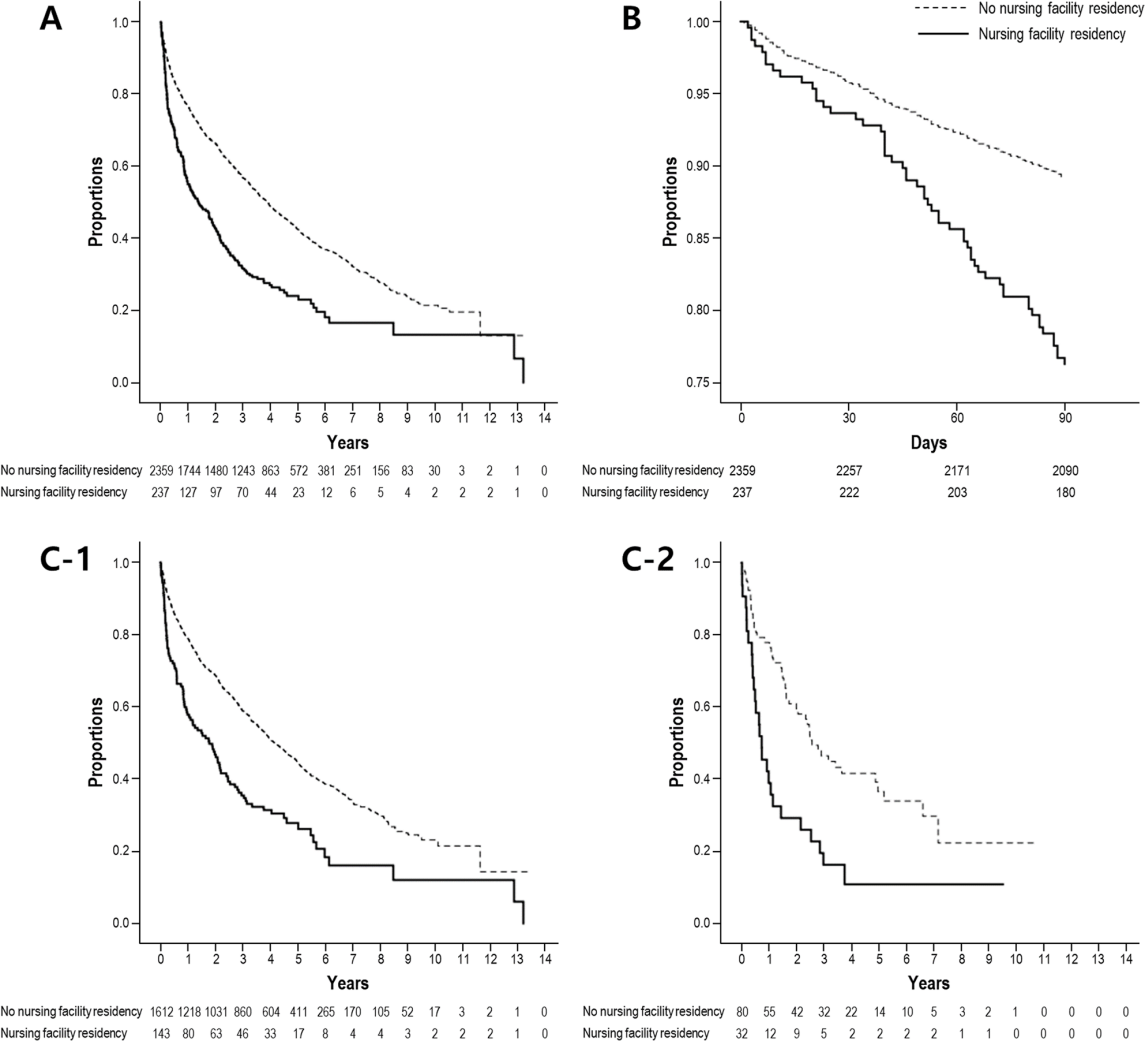
Survival analysis based on nursing facility residency status. Survival rates are presented for two patient groups—those with and without a history of nursing facility residency—using Cox regression survival curves. The dashed line represents patients without a history of nursing facility residency, and the solid line represents those with nursing facility residency. The vertical axis indicates the proportion of surviving patients, and the horizontal axis indicates the time elapsed in days. Patients in the nursing facility residency group demonstrated a notably faster decline in survival rates compared with those without nursing facility residency. **A** Overall survival during the entire follow-up period. The accompanying table below the curves indicates patient counts over time, beginning with 2,359 patients in the group without nursing facility residency and 237 patients in the nursing facility residency group (*p* < 0.001). **B** Early survival within the first 90 days after dialysis initiation. **C** Subgroup analyses stratified by dementia status: (C-1 patients without dementia; (C-2) patients with dementia

The survival analysis curves using Cox regression, comparing patients with and without nursing facility residency, are shown in Fig. 2A. The initial patient counts were 237 in the nursing facility residency group and 2,359 in the non-residency group. The median follow-up duration was 2,167 days (95% CI, 2,071–2,263), estimated using the reverse Kaplan–Meier method. 1,595 out of total 2,597 patients died. The survival curve for the nursing facility group declined more rapidly, consistently demonstrating a lower proportion of surviving patients. Mortality rate (deaths per 100 person-years) in nursing facility group was 40.2 (total death: 1,760; total person-years: 7,859.86) while that of without nursing facility residency group was 22.4 (total death: 199; total person-years: 495.58). Cumulative survival at pre-specified time points in nursing facility group were 43%, 27%, 18% at 1, 3, 5 years respectively; those of without nursing facility residency group were 66%, 49%, 37%. This indicates a higher mortality rate in the group with nursing facility residency, suggesting that residing in a nursing facility could significantly influence mortality risk.

Multivariate Cox regression analysis results (Table 4), after adjusting for demographic characteristics, laboratory findings, and medication use, identified several factors associated with increased mortality. Specifically, dementia (adjusted hazard ratio [HR]: 1.550, 95% CI: 1.102–2.180; *p* = 0.012), malignancy (HR: 1.256, 95% CI: 1.074–1.469; *p* = 0.004), and depression drug (HR: 1.395, 95% CI: 1.074–1.811; *p* = 0.012) were significantly associated with higher mortality rates among HD patients. Most importantly, having a history of residency in a nursing facility showed the highest hazard ratio (HR: 1.659, 95% CI: 1.297–2.216; *p* < 0.001), strongly suggesting that nursing facility residency itself substantially influenced the increased mortality observed in these HD patients.

**Table 4:** Multivariate Cox regression model of nursing facility residency

Variables	Univariate HR [95% CI]	*P* value of univariate model	Multivariate HR [95% CI]	*P* value of multivariate model
Nursing facility residency	1.851 [1.584, 2.164]	< 0.001	1.695 [1.297, 2.216]	< 0.001
Age at dialysis	1.049 [1.040, 1.058]	< 0.001	1.052 [1.041, 1.063]	< 0.001
Sex	0.906 [0.823, 0.997]	0.044	0.776 [0.654, 0.922]	0.004
Dementia	1.465 [1.157, 1.856]	0.002	1.550 [1.102, 2.180]	0.012
AVF when starting dialysis	0.766 [0.712, 0.824]	< 0.001	0.813 [0.725, 0.911]	< 0.001
Malignancy	1.331 [1.214, 1.460]	< 0.001	1.256 [1.074, 1.469]	0.004
Ischemic heart disease	1.157 [1.031, 1.299]	0.013	1.003 [0.820, 1.228]	0.974
Cerebrovascular disease	1.184 [1.053, 1.332]	0.005	1.142 [0.934, 1.396]	0.197
Heart failure	1.329 [1.178, 1.499]	< 0.001	1.193 [0.962, 1.478]	0.107
Atrial fibrillation	1.509 [1.299, 1.753]	< 0.001	1.173 [0.872, 1.579]	0.291
Liver cirrhosis	1.571 [1.221, 2.021]	< 0.001	1.227 [0.768, 1.960]	0.391
Fracture	1.103 [1.011, 1.204]	0.028	1.109 [0.861, 1.428]	0.422
WBC (/mm^3^)	1.000 [1.000, 1.000]	0.007	1.000 [1.000, 1.000]	0.361
Platelet (/mm^3^)	1.000 [1.000, 1.000]	0.012	1.000 [1.000, 1.000]	0.827
Creatinine (mg/dL)	0.915 [0.897, 0.933]	< 0.001	0.955 [0.925, 0.987]	0.006
Albumin (g/dL)	0.603 [0.556, 0.653]	< 0.001	0.852 [0.747, 0.972]	0.017
Phosphorus (mg/dL)	0.944 [0.915, 0.973]	< 0.001	1.035 [1.000, 1.072]	0.052
Total cholesterol (mg/dL)	0.998 [0.997, 0.999]	< 0.001	1.000 [0.998, 1.002]	0.954
RAAS blockade	0.715 [0.649, 0.787]	< 0.001	0.870 [0.738, 1.026]	0.097
Beta blocker	0.892 [0.808, 0.984]	0.023	1.012 [0.855, 1.198]	0.888
Calcium channel blocker	0.680 [0.618, 0.748]	< 0.001	0.735 [0.622, 0.867]	< 0.001
Warfarin	1.501 [1.192, 1.890]	0.001	1.005 [0.647, 1.561]	0.982
Calcium-based phosphate binder	0.747 [0.676, 0.826]	< 0.001	0.857 [0.727, 1.010]	0.066
Sevelamer	0.581 [0.424, 0.795]	0.001	0.532 [0.329, 0.862]	0.010
Depression drug	1.254 [1.064, 1.477]	0.007	1.395 [1.074, 1.811]	0.012

For continuous laboratory variables, hazard ratios represent the effect per 1-unit increase in the original measurement scale (e.g., mg/dL for creatinine)

*Abbreviations*: *AVF* Arteriovenous fistula, *WBC* White blood cell, *RAAS* Renin-angiotensin-aldosterone system

To evaluate whether the observed survival difference was influenced by early deaths occurring shortly after dialysis initiation, we performed Kaplan–Meier analyses restricted to the first 90 days of follow-up. During this early period, patients residing in nursing facilities at the time of dialysis initiation exhibited significantly lower survival compared with those without nursing facility residency (Fig. 2B). In the analysis focusing on early mortality within 90 days after dialysis initiation (Table 5), nursing facility residency remained independently associated with a significantly increased risk of death after multivariable adjustment (adjusted HR: 2.379, 95% CI: 1.516–3.734; *p* < 0.001), indicating that the excess mortality risk was not confined to long-term follow-up but was already evident during the early dialysis period.

**Table 5:** Multivariate Cox regression model of nursing facility residency of 90 days survival rate

Variables	90 Days Survival Rate [95% CI]	*P* value
Nursing facility residency	2.379 [1.516, 3.734]	< 0.001
Age at dialysis	1.051 [1.020, 1.084]	0.001
Sex	1.103 [0.763, 1.595]	0.601
Dementia	0.831 [0.408, 1.692]	0.610
AVF when starting dialysis	0.751 [0.532, 1.061]	0.104
Malignancy	1.529 [1.169, 1.999]	0.002
Ischemic heart disease	1.360 [0.900, 2.055]	0.144
Cerebrovascular disease	0.989 [0.628, 1.558]	0.963
Heart failure	1.109 [0.720, 1.708]	0.639
Atrial fibrillation	1.082 [0.623, 1.880]	0.779
Liver cirrhosis	2.526 [1.251, 5.099]	0.010
Fracture	1.398 [0.917, 2.133]	0.120
WBC (/mm^3^)	1.000 [1.000, 1.000]	0.101
Platelet (/mm^3^)	1.000 [1.000, 1.000]	0.265
Creatinine (mg/dL)	0.903 [0.834, 0.978]	0.012
Albumin (g/dL)	0.594 [0.444, 0.794]	< 0.001
Phosphorus (mg/dL)	1.026 [0.967, 1.088]	0.395
Total cholesterol (mg/dL)	1.001 [0.997, 1.004]	0.650
RAAS blockade	0.598 [0.408, 0.878]	0.009
Beta blocker	0.646 [0.432, 0.965]	0.033
Calcium channel blocker	0.649 [0.451, 0.934]	0.020
Warfarin	0.763 [0.350, 1.662]	0.496
Calcium-based phosphate binder	0.601 [0.407, 0.888]	0.010
Sevelamer	1.132 [0.444, 2.883]	0.795
Depression drug	0.998 [0.541, 1.841]	0.996

For continuous laboratory variables, hazard ratios represent the effect per 1-unit increase in the original measurement scale (e.g., mg/dL for creatinine)

*Abbreviations*: *AVF* Arteriovenous fistula, *WBC* White blood cell, *RAAS* Renin-angiotensin-aldosterone system

In addition, to assess the robustness of the association across clinically relevant subgroups, we conducted stratified Kaplan–Meier analyses according to dementia status. In both patients without dementia and those with dementia, nursing facility residency at dialysis initiation was consistently associated with lower survival throughout follow-up (Fig. 2C-1 and 2C-2). Although absolute survival differed between subgroups, the overall pattern of reduced survival among nursing facility residents was preserved. Dementia-stratified multivariable analyses demonstrated that nursing facility residency was consistently associated with higher mortality risk in both patients with and without dementia (HR: 3.301, 95% CI: 1.597–6.826; *p* = 0.001; HR: 1.568, 95% CI: 1.231–1.998; *p* < 0.001, respectively) (Table 6). Although the magnitude of the association differed between subgroups, the direction of effect remained uniform, suggesting that the association between nursing facility residency and mortality was not driven solely by cognitive impairment.

**Table 6:** Multivariate Cox regression model of nursing facility residency according to dementia diagnosis

Variables	No dementia [95% CI]	*P* value of no dementia	Dementia [95% CI]	*P* value of dementia
Nursing facility residency	1.568 [1.231, 1.998]	< 0.001	3.301 [1.597, 6.826]	0.001
Age at dialysis	1.063 [1.049, 1.077]	< 0.001	1.112 [1.043, 1.187]	0.001
Sex	1.312 [1.126, 1.528]	< 0.001	0.334 [0.162, 0.688]	0.003
AVF when starting dialysis	0.848 [0.765, 0.940]	0.002	1.471 [0.560, 3.865]	0.434
Malignancy	1.336 [1.158, 1.541]	< 0.001	0.789 [0.404, 1.538]	0.486
Ischemic heart disease	1.075 [0.900, 1.285]	0.425	4.847 [1.808, 12.989]	0.002
Cerebrovascular disease	1.210 [1.006, 1.454]	0.043	1.483 [0.677, 3.245]	0.324
Heart failure	1.316 [1.087, 1.592]	0.005	0.441 [0.182, 1.071]	0.070
Atrial fibrillation	1.151 [0.885, 1.496]	0.293	0.624 [0.171, 2.272]	0.474
Liver cirrhosis	1.483 [1.016, 2.165]	0.041	0.000 [0.000, 0.000]	0.979
Fracture	1.130 [0.923, 1.383]	0.236	1.145 [0.345, 3.800]	0.825
WBC (/mm^3^)	1.000 [1.000, 1.000]	0.757	1.000 [1.000, 1.000]	0.509
Platelet (/mm^3^)	1.000 [1.000, 1.000]	0.712	1.000 [1.000, 1.000]	0.077
Creatinine (mg/dL)	0.965 [0.938, 0.994]	0.019	0.989 [0.826, 1.184]	0.902
Albumin (g/dL)	0.758 [0.672, 0.855]	< 0.001	0.671 [0.385, 1.166]	0.157
Phosphorus (mg/dL)	1.013 [0.975, 1.052]	0.512	1.309 [1.078, 1.589]	0.007
Total cholesterol (mg/dL)	1.000 [0.999, 1.002]	0.586	0.992 [0.983, 1.001]	0.088
RAAS blockade	0.876 [0.758, 1.012]	0.072	2.431 [1.029, 5.744]	0.043
Beta blocker	0.939 [0.806, 1.094]	0.419	0.803 [0.373, 1.733]	0.577
Calcium channel blocker	0.777 [0.670, 0.902]	0.001	0.737 [0.328, 1.652]	0.458
Warfarin	0.973 [0.663, 1.428]	0.888	0.648 [0.161, 2.601]	0.540
Calcium-based phosphate binder	0.837 [0.721, 0.971]	0.019	0.431 [0.184, 1.012]	0.053
Sevelamer	0.619 [0.389, 0.985]	0.043	0.556 [0.098, 3.144]	0.507
Depression drug	1.122 [0.885, 1.424]	0.342	1.654 [0.514, 5.321]	0.399

For continuous laboratory variables, hazard ratios represent the effect per 1-unit increase in the original measurement scale (e.g., mg/dL for creatinine)

*Abbreviations*: *AVF* Arteriovenous fistula, *WBC* White blood cell, *RAAS* Renin-angiotensin-aldosterone system

## Discussion

Given the specific context of Korea, where the population of older adults receiving hemodialysis continues to increase, it is essential to investigate characteristics associated with mortality in this setting. This retrospective study suggests that nursing facility residency may be associated with increased mortality among older Korean ESRD patients. While prior studies from Europe and the United States have demonstrated high mortality and functional decline among elderly dialysis patients [13–15], data from Asian populations remain limited. Our findings are directionally consistent with international reports indicating increased vulnerability among institutionalized or functionally dependent older dialysis patients, although differences in healthcare systems and long-term care infrastructure should be considered when interpreting cross-national comparisons.

Several limitations should be acknowledged. As a retrospective observational study, residual confounding and reverse causation cannot be fully excluded, particularly given the lack of direct measures of frailty and functional status. Nursing facility residence likely reflects underlying vulnerability rather than an independent causal effect. These issues are discussed in detail in the Limitations section below.

Differences in healthcare systems, long-term care structures, and patient selection criteria between Western countries and Korea may further contribute to variations in effect size. In addition, prior studies frequently examined broader or more granular definitions of institutional residence, whereas our exposure was defined as nursing facility residency at the time of dialysis initiation, potentially yielding a more conservative risk estimate. In this context, Chen et al. incorporated longitudinal residence histories and distinguished between short- and long-term nursing home stays using claims-based data, which may partly explain the higher effect estimates observed in their cohort. Indeed, these findings are also consistent with recent national registry data from Korea showing persistently high early mortality among older hemodialysis patients despite overall improvements in long-term survival, suggesting that vulnerability at dialysis initiation remains a critical determinant of outcomes [3].

In the present study, patients residing in nursing facilities were more frequently prescribed antiplatelet agents, warfarin, medications for dementia, and antidepressants compared with community-dwelling patients. These prescribing patterns likely reflect a higher burden of comorbid conditions, such as cerebrovascular disease, atrial fibrillation, cognitive impairment, and depressive symptoms, rather than intentional differences in treatment strategy. Although our dataset did not include the total number of concurrently prescribed medications, previous studies have reported a high prevalence of polypharmacy among patients with chronic kidney disease, particularly those receiving dialysis [16]. Prior literature has suggested that the use of multiple medications, including psychotropic and antithrombotic agents, may be associated with adverse outcomes such as falls, bleeding events, and increased mortality in older dialysis populations [17, 18]. Therefore, the observed differences in medication classes in nursing facility residents should be interpreted as potential markers of clinical complexity and vulnerability, rather than as direct evidence of polypharmacy-driven mortality.

Indeed, Baseline standardized mean differences demonstrated that nursing facility residents were clinically distinct at dialysis initiation, particularly with respect to dementia, sex distribution, cerebrovascular disease, and body weight. These differences support the interpretation that facility residence reflects a more vulnerable population. Although multivariable adjustment was performed, residual imbalance may persist.

In our cohort, patients with a history of nursing facility residency exhibited laboratory and clinical features suggestive of poorer nutritional and health status, including lower serum albumin, phosphorus, total cholesterol, and lymphocyte counts, as well as a higher prevalence of comorbidities such as dementia, heart failure, malignancy, and cerebrovascular disease. These findings may overlap with certain features commonly observed in malnutrition–inflammation complex syndrome (MICS), which has been associated with adverse outcomes in patients with chronic kidney disease [17–20] .

However, given the absence of direct inflammatory markers and standardized diagnostic criteria for MICS in our dataset, this interpretation should be considered hypothesis-generating rather than confirmatory. Nursing facility residence may therefore reflect a constellation of vulnerability-related factors, including nutritional insufficiency and comorbidity burden, rather than a specific pathophysiological mechanism. Additional factors such as infection risk and falls, which have been reported to be more common among older nursing facility residents and patients receiving hemodialysis [21, 22], may further contribute to adverse outcomes in this population.

Moreover, it remains important to consider whether nursing facility residency represents an independent factor associated with mortality or whether it serves as an intermediate marker reflecting previously identified vulnerabilities, such as frailty or diminished functional status [23]. Frailty is highly prevalent among older adults receiving dialysis and is independently associated with increased mortality. Nursing facility residence at dialysis initiation may serve as a surrogate marker of frailty and functional dependence, partially explaining the observed association. However, given the complex and interrelated nature of these factors, nursing facility residency should be interpreted primarily as a clinical context in which multiple risk factors may coexist. In this regard, greater attention to individualized care needs and advance care planning may be warranted for older patients initiating hemodialysis while residing in nursing facilities, with the aim of supporting patient-centered decision-making and overall well-being.

## Limitations

This study has several limitations due to its retrospective nature, providing associative evidence rather than demonstrating clear causality, and difficulty fully controlling potential confounding factors such as frailty, functional status, and social vulnerability. Nursing facility residence at dialysis initiation likely reflects a complex constellation of functional dependence, cognitive impairment, reduced social support, and overall frailty—factors that are themselves strong predictors of mortality. Because our dataset did not include standardized assessments of activities of daily living, formal frailty indices (e.g., Fried criteria or SPPB), or detailed measures of social determinants of health, substantial residual confounding is likely. Therefore, the observed association may represent underlying patient vulnerability rather than an independent causal effect of residence status per se, and the magnitude of association may be attenuated with more comprehensive adjustment.

Also, the use of complete-case analysis may have introduced selection bias and should be considered when interpreting the results. Furthermore, an imbalance in the follow-up period—mortality data provided by the Korean National Statistical Office extended only up to 2020, while patient data enrolled from KSGN were available only up to 2017—could introduce statistical errors or bias. Moreover, several sensitivity and subgroup analyses, such as stratification by age, sex, or nutritional markers, as well as landmark analyses excluding early mortality, would have provided additional insight into the robustness of our findings. However, the retrospective nature of the dataset and limitations in data availability precluded reliable implementation of these analyses. In particular, detailed longitudinal information on changes in clinical status and sufficiently complete data for subgroup stratification were not available across all participating centers. Hence, although we included serum albumin, phosphorus, total cholesterol, and lymphocyte counts as indirect markers of nutritional and inflammatory status, these measures do not fully capture nutritional reserve or sarcopenia in older dialysis patients. Reverse causality may also have contributed to the observed association, as patients experiencing acute clinical deterioration may have been more likely to reside in nursing facilities at the time of dialysis initiation.

Certain medication variables, including antidepressants and dementia-related therapies, were included as proxies of baseline clinical complexity. However, these factors may also lie along the pathway between frailty and mortality, raising the possibility of over-adjustment or collider bias. Therefore, the estimated hazard ratios should be interpreted with caution. Moreover, laboratory parameters were obtained prior to or at dialysis initiation; however, differences in residual renal function between groups may have influenced these measurements. Such variability could introduce additional residual confounding that cannot be fully accounted for in this retrospective design.

Accordingly, residual confounding and reverse causation—whereby patients with severe baseline illness were more likely to reside in nursing facilities and experience early mortality—cannot be fully excluded. Our findings should therefore be interpreted as demonstrating an association rather than a causal relationship. Future prospective studies with standardized data collection and predefined sensitivity analyses are warranted to more rigorously address these issues.

Patients residing in nursing facilities may differ from those living at home in several unmeasured aspects, including functional dependence, nutritional status, and overall intensity of care. Although our database did not include direct measures of psychosocial support, frailty, or medication burden, these factors may partially explain the observed association between nursing-facility residence and mortality. In addition, information on timing of nephrology referral and pre-dialysis nephrology care and socioeconomic status was unavailable, and residual confounding by unmeasured factors cannot be fully excluded. Furthermore, our exposure definition was based on residence status at the time of dialysis initiation and did not include information regarding the duration of nursing facility stay. Therefore, we were unable to distinguish between long-term residents with chronic disability and patients who were recently admitted due to acute clinical deterioration. These groups likely differ substantially in baseline health status and short-term mortality risk. This temporal ambiguity may have contributed to reverse causation, particularly if patients experiencing rapid clinical decline were more likely to initiate dialysis while residing in a facility.

As an exploratory robustness assessment, the E-value for the observed adjusted hazard ratio (1.695) was 2.78, and the E-value for the lower bound of the confidence interval (1.297) was 1.92. These findings suggest that an unmeasured confounder associated with both nursing facility residence and mortality by a risk ratio of at least 2.78 each, beyond measured covariates, would be required to fully explain away the observed association. Nevertheless, nursing-facility residence remained statistically associated with mortality after adjustment for measured covariates; however, given the absence of direct frailty and functional assessments, the magnitude and independence of this association should be interpreted cautiously. Future studies incorporating standardized assessments of frailty and social determinants of health may help clarify underlying mechanisms.

### Clinical implications

From a clinical perspective, our findings highlight the importance of heightened clinical vigilance for older patients initiating hemodialysis while residing in nursing facilities. Rather than serving as a determinant for dialysis initiation or withdrawal decisions, nursing facility residence should be viewed as a contextual factor that may signal increased vulnerability. In this setting, systematic nutritional screening, regular medication review with attention to potentially inappropriate prescribing, and structured assessment of functional status and frailty may help identify patients at higher risk of adverse outcomes.

Importantly, decisions regarding dialysis initiation and ongoing management should not be based solely on the care setting. Instead, our results underscore the need for a multidisciplinary approach involving nephrologists, geriatric specialists, nursing staff, patients, and caregivers, with an emphasis on shared decision-making that aligns treatment plans with individual health status, goals of care, and patient preferences.

## Conclusions

In this multicenter retrospective cohort study, residence in a nursing facility at the time of dialysis initiation was associated with higher mortality among older patients receiving hemodialysis. These findings suggest that residence status may serve as a clinically relevant marker of vulnerability in this population.

Given the observational design and the lack of data on certain clinical and social factors, causal inferences cannot be drawn. Future prospective studies incorporating standardized assessments of frailty and social determinants of health are needed to better understand the mechanisms underlying this association.

## Data Availability

The datasets used and/or analysed during the current study are available from the corresponding author on reasonable request.

## Abbreviations

AF: Atrial fibrillation
ALP: Alkaline phosphatase
BUN: Blood urea nitrogen
CAD: Coronary artery disease
CI: Confidence interval
CKD: Chronic kidney disease
CVD: Cerebrovascular disease
DM: Diabetes mellitus
EF: Ejection fraction
ESRD: End-stage renal disease
HD: Hemodialysis
HF: Heart failure
HR: Hazard ratio
iPTH: Intact parathyroid hormone
IRB: Institutional Review Board
KORDS: Korean Renal Data System
KSN: Korean Society of Nephrology
KSGN: Korean Society of Geriatric Nephrology
MICS: Malnutrition-inflammation complex syndrome
PAD: Peripheral artery disease
